# Exploring predictors of post-COVID-19 condition among 810 851 individuals in Sweden

**DOI:** 10.1038/s43856-025-01157-2

**Published:** 2025-10-30

**Authors:** Yiyi Xu, Huiqi Li, Robert Sigström, Lisa Lundberg-Morris, Magnus Gisslén, Simon B. Larsson, Fredrik Nyberg, Maria Bygdell

**Affiliations:** 1 School of Public Health and Community Medicine, Sahlgrenska Academy, University of Gothenburg, Gothenburg, Sweden; 2 Department of Psychiatry and Neurochemistry, Institute of Neuroscience and Physiology, Sahlgrenska Academy, University of Gothenburg, Gothenburg, Sweden; 3 Department of Psychiatry Affective Disorders, Sahlgrenska University Hospital, Region Västra Götaland, Gothenburg, Sweden; 4 Department of Microbiology and Immunology, Institute of Biomedicine, Sahlgrenska Academy, University of Gothenburg, Gothenburg, Sweden; 5 Unit of Clinical Pharmacology, Department of Pharmaceuticals, Sahlgrenska University Hospital, Region Västra Götaland, Gothenburg, Sweden; 6 Department of Infectious Diseases, Institute of Biomedicine, Sahlgrenska Academy, University of Gothenburg, Gothenburg, Sweden; 7 Department of Infectious Diseases, Sahlgrenska University Hospital, Region Västra Götaland, Gothenburg, Sweden; 8 Public Health Agency of Sweden, Solna, Sweden; 9 Department of Addictions, Sahlgrenska University Hospital, Region Västra Götaland, Gothenburg, Sweden; 10 Department of Internal Medicine and Clinical Nutrition, Sahlgrenska Academy, University of Gothenburg, Gothenburg, Sweden

**Keywords:** Viral infection, Epidemiology

## Abstract

**Background:**

Long-term effects of COVID-19 can place burden on individuals, healthcare, and society. We aimed to evaluate the importance of a wide range of potential risk factors for being diagnosed with post-COVID-19 condition (PCC).

**Methods:**

We used data from national and regional registers and databases for all adult residents in the two largest regions in Sweden. Individuals with a first COVID-19 between 1 August 2020 and 9 February 2022 were included and followed until PCC diagnosis, censoring (death or migration), or 30 November 2023. Using Cox proportional hazards models and backwards stepwise selection, we evaluated a large set of risk factors including sociodemographic data, comorbidities, healthcare contact behaviors, COVID-19-related factors, as well as PCC in family and cohabitants (as proxies for genetics and shared environment).

**Results:**

We include 810,851 individuals (age range 18-106 years and 53.3% women), of whom 1.4% are diagnosed with PCC during follow-up. Female sex, older age, being born outside Sweden, higher educational attainment, essential workers, having comorbidities such as thromboembolic disease, asthma, fibromyalgia, depression/anxiety, and stress-related disorders, being infected earlier in the study period, experiencing severe acute COVID-19, not being vaccinated before COVID-19, and having a relative or a cohabitant with PCC are associated with an increased risk of being diagnosed with PCC.

**Conclusions:**

In this large population-based cohort study, our exploratory analysis reveals several risk factors for being diagnosed with PCC. Our findings can serve as a basis for future targeting of preventive measures against PCC.

## Plain language summary

Long-term effects of COVID-19 can place burden on individuals, healthcare, and society. We aimed to explore a wide range of potential risk factors for being diagnosed with post-COVID-19 condition (PCC). We included 810,851 individuals, of whom 1.4% were diagnosed with PCC during follow-up, using data from registers in Sweden. Female sex, older age, being born outside Sweden, higher educational attainment, essential workers, having comorbidities such as thromboembolic disease, asthma, fibromyalgia, depression/anxiety, and stress-related disorders, being infected earlier in the study period, experiencing severe acute COVID-19, not being vaccinated before COVID-19, and having a relative or a cohabitant with PCC are associated with an increased risk of being diagnosed with PCC. Our findings can serve as a basis for future targeting of preventive measures against PCC.

## Abstract (web summary)

Xu et al. explore predictors of post-COVID-19 condition (PCC) among 810,851 individuals in Sweden using register data. The results reveal several risk factors for being diagnosed with PCC that can serve as a basis for future targeting of preventive measures against PCC.

## Introduction

COVID-19 has been a worldwide challenge and an acute threat to individuals and public health for several years. Although the World Health Organization (WHO) no longer considers COVID-19 a threat of international concern, the long-term effects of the disease can still be a burden on individuals and continue to strain healthcare and society as a whole^1^. In September 2020, WHO introduced an International Classification of Diseases 10^th^ revision (ICD-10) diagnosis code (U09.9)^2^ for long-term sequelae from COVID-19, also called long COVID and post-COVID-19 condition (PCC)^3^. In Sweden, this diagnosis code was implemented already in October 2020^4^. The National Board of Health and Welfare states that this code should be used to describe a condition or symptom that a physician assesses to be caused by a previous SARS-CoV-2 infection^4^. This fast implementation in combination with the large healthcare registers in Sweden enables comprehensive epidemiological research on the use and occurrence of the PCC diagnosis code, in the whole population.

As the virus causing COVID-19, SARS-CoV-2, will continue circulating in the population for the coming years, infections and reinfections will remain common. Although the incidence of PCC appears to be decreasing, likely due to increased immunity against SARS-CoV-2, identifying individuals at elevated risk for long-term complications after infection is a high priority. In a public health perspective, appropriate targeting of preventive measures, as well as early detection for better disease management, will benefit from research clearly identifying which groups in society are at highest risk of experiencing long-term effects. To date, risk factors that have been identified to associate with PCC include female sex^5–11^, older age^5–10,12^, comorbidities such as asthma, chronic obstructive pulmonary disease (COPD), and high Body Mass Index (BMI)^7–12^, as well as severe acute COVID-19^5,8,10,12,13^. Factors associated with a decreased risk for PCC have been vaccination^6,8,12,14,15^ and infection by the Omicron vs earlier variants^5,6,11,16–18^. Previous studies evaluating risk factors for PCC have often been small and relied on selected patient populations, resulting in study populations not reflective of the total population at risk. A recent systematic review with a meta-analysis concluded that female sex, older age, high BMI, smoking, certain comorbidities, and hospitalization for acute COVID-19 were associated with an increased risk for PCC, while vaccination was associated with a decreased risk^19^.

Using a large and rich population-based dataset with a source population covering approximately 40% of the Swedish population, we have a unique opportunity to address several remaining questions regarding risk factors for PCC. The aim of the present study was therefore to perform an explorative risk factor analysis covering a wide range of factors for the risk of receiving a diagnosis of PCC after the first SARS-CoV-2 infection in the total population. We show that female sex, older age, being born outside Sweden, higher educational attainment, essential workers, having comorbidities such as thromboembolic disease, asthma, fibromyalgia, depression/anxiety, and stress-related disorders, being infected earlier in the study period, experiencing severe acute COVID-19, not being vaccinated before COVID-19, and having a relative or a cohabitant with PCC are associated with an increased risk of being diagnosed with PCC.

## Methods

### Study design and data sources

This population-based cohort study is part of the SCIFI-PEARL project (Swedish Covid-19 Investigation for Future Insights – a Population Epidemiology Approach using Register Linkage), which is a nationwide multi-register, regularly updated, observational study of the COVID-19 pandemic in Sweden^20^. The SCIFI-PEARL project links a broad range of national and regional healthcare registers using the unique Swedish personal identification number^21^ and forms a pseudonymized dataset. In the present study, data on positive SARS-CoV-2 polymerase chain reaction (PCR) test results were obtained from the National Register of Notifiable Diseases (SmiNet). Diagnoses of COVID-19, PCC, and comorbidities were retrieved from the National Patient Register (NPR), including both specialist inpatient care and outpatient visits, and from two regional databases of all public and most private primary healthcare (VAL and VEGA, in Region Stockholm and Region Västra Götaland, respectively) using the ICD-10 Swedish version (ICD-10-SE) diagnosis codes. Prescriptions of medication were retrieved from the National Prescribed Drug Register. Additional data on COVID-19-related intensive care unit (ICU) stays were obtained from the Swedish Intensive Care Register. Data on COVID-19 vaccination were retrieved from the National Vaccination Register (NVR), and data on death, emigration, demographic, and socioeconomic data from the Total Population Register and the Longitudinal Integrated Database for Health Insurance and Labour Market Studies (LISA). Information on kinship was retrieved from the Multigeneration Register and information on cohabitation was retrieved from the Apartment register. The study was approved by the Swedish Ethical Review Authority (Dnr: 2020-01800 with several amendments) who waived the requirement of informed consent. Data were obtained from the register holders after approved applications.

### Study population, cohort definition, and follow-up

**Fig. 1:**
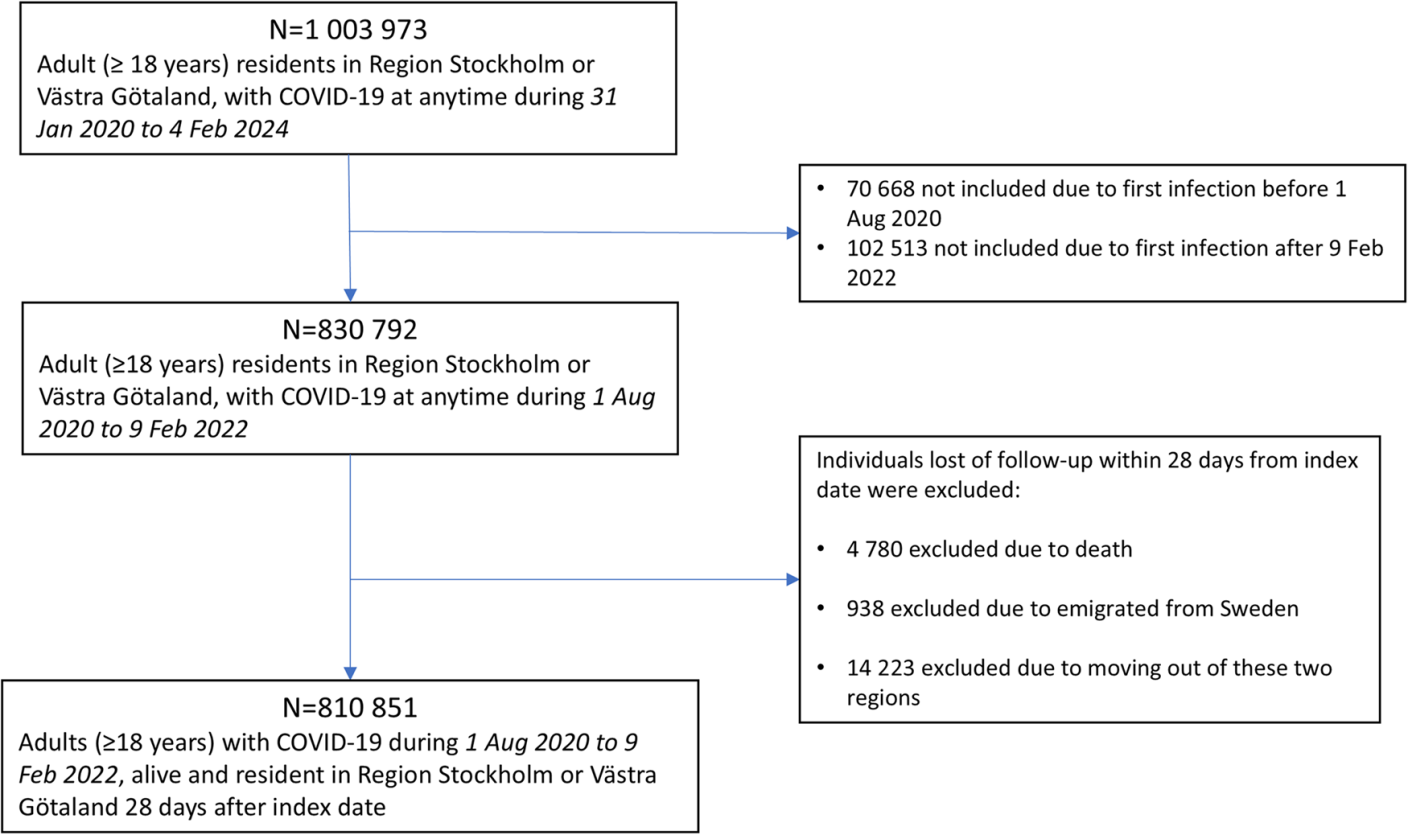
Flowchart of the study population. Selection process of the study population for the risk factor analysis of post-COVID-19 condition (PCC) in all individuals with COVID-19 in the two largest regions of Sweden.

As PCC mainly is diagnosed in primary healthcare in Sweden^22^ and we have access to data from the primary healthcare databases VAL and VEGA, this study included all residents from these two regions (about 40% of the Swedish population) who were registered with their first COVID-19 (based on either a positive PCR test [83%] or ICD-10-SE diagnosis codes U07.1 or U07.2 as main or secondary diagnosis) during the extensive PCR testing period in Sweden (i.e., 1 August 2020 to 9 February 2022) and were ≥18 years of age at time of registered infection. All individuals with any symptom of ongoing COVID-19 were recommended to take a PCR test during this period, which was easy to access and free of charge. Date of registered COVID-19 was considered the index date, and every individual was followed with start from 28 days after index date until a valid PCC diagnosis (see Outcome section), death, emigration, move out from the two regions, or end of the study (30 November 2023), whichever came first. Individuals who died, emigrated, or moved out from the two regions within 28 days after index date were not at risk of PCC as captured in the study and were thus not included. Finally, 810 851 individuals were included in the study cohort (Fig. 1). In a sensitivity analysis, 90 days interval between index date and a valid PCC diagnosis was used instead of 28 days, resulting in 807 517 individuals in the study cohort.

### Outcome - PCC

A valid clinical diagnosis for PCC was defined as ICD-10-SE code U09.9 in NPR, VEGA, or VAL as the main or secondary diagnosis ≥28 days after index date. A minimum of 28 days between index date and PCC diagnosis was required, as a PCC diagnosis within 28 days was interpreted as a likely misclassification relating to health effects of the acute infection rather than PCC. In the sensitivity analysis, we used a 90-day interval between index date and a valid PCC diagnosis^3^.

### Evaluated risk factors

A predetermined list of potential risk factors of interest was selected by literature review and clinical knowledge and included factors reflecting personal sociodemographic data, health conditions, healthcare contact behaviors, COVID-19-related factors, area-level socioeconomic status (SES), as well as PCC in family and cohabitants (as proxies for genetic and shared environmental factors).

Personal sociodemographic factors included: age at first infection (categorized as 18-39, 40-79, and ≥80 years of age due to a non-linear association with the outcome [Supplementary Fig. 1]), sex (women, men), country of birth (Sweden, non-Sweden), parents’ country of birth (both from Sweden, one from Sweden, both non-Sweden, and unknown), education levels (primary [≤9 years], upper secondary [10-12 years], tertiary [≥13 years], and unknown), income levels (four quartile groups based on the distribution of disposable income per consumption unit in the study cohort), occupation (healthcare workers, other essential workers [including teachers, service sector workers, police and security services, postal and delivery workers, cleaners, and taxi, bus, and tram drivers], non-essential workers, and unemployed), marital status (married, not married), and region of residence (Region Stockholm, Region Västra Götaland). All personal sociodemographic factors, except age, were obtained before the pandemic, i.e., at the end of 2019.

Personal health conditions were defined based on specific comorbidities during 2015-2019 and medication use during 2018-2019. Comorbidities included cardiovascular diseases (heart failure, ischemic heart diseases, stroke, peripheral vascular disease, thromboembolic disease, arrythmias, and other cardiac diseases); respiratory diseases (COPD, asthma, and other respiratory diseases), metabolic syndrome components (hypertension, type 2 diabetes), chronic kidney disease, immune disorders and immune suppression, autoimmune diseases, fibromyalgia, and psychiatric disorders (dementia, bipolar disorder and schizophrenia, depression and anxiety, stress-related disorders, and other mental disorders). The ICD-10-SE and Anatomical Therapeutic Chemical Classification System (ATC) codes defined these conditions (Supplementary Table 1). The total number of these specific comorbidities for each individual observed in 2015-2019 was additionally calculated and grouped into three categories (0, 1-2, and ≥3). Number of healthcare (primary and specialist care) contacts in 2019 (categorized as 0, <10, and ≥10 according to a non-linear association with the outcome [Supplementary Fig. 2]) was used as a surrogate of personal healthcare seeking behaviors.

COVID-19-related factors included: variants of concern (VOC) period when particular virus variants were dominating as the first infection occurred (preAlpha, Alpha, Delta, and Omicron [Supplementary Table 2]), severity of first acute infection (non-hospitalized, hospitalization without ICU, and ICU admission), and any vaccination ≥14 days before first infection (no, yes).

Area-level SES was calculated as the proportion of inhabitants in each specific geographical area (Demographic Statistical Areas, DeSO)^23^ with an annual income lower than the first quartile of the national average income level in 2019 and further categorized into low (>0.4; i.e. 40% of inhabitants with low annual income) or high (≤0.4) area-level SES according to a non-linear association with the outcome (Supplementary Fig. 3). Thus, study individuals living in the same specific geographical area will have the same area-level SES.

We also considered PCC diagnosis in family and cohabitants as potential risk factors, defined as: PCC cases at any time in the core family (yes, no or uncertain [family members not living in the included regions]), and PCC cases at any time in cohabitants (yes, no or uncertain). The core family was defined as biological parents and children.

### Statistics and reproducibility

Descriptive analysis (count and proportion) for each risk factor is presented according to PCC status at the end of follow-up. Penalized smoothing splines was used to illustrate and determine the appropriate categories for age, number of healthcare contacts in 2019, and area-level SES, using R package “pspline”. Crude incidence rates of PCC (per 1000 person-years) are presented in each variable category. Cox proportional hazards models, with time-on-study as the underlying time scale, were used to estimate hazard ratio (HR) with 95% confidence interval (95%CI). First, separate crude univariable models were run for each risk factor, then a full model including all risk factors simultaneously was run. To identify the most important risk factors, backwards stepwise selection was applied, starting with the full model. Factors were removed if their significance level was p ≥ 0.1 and reintroduced if p ≤ 0.05. To access the robustness of the variable selection, LASSO regression using Bayesian Information Criterion (BIC) and adaptive methods was conducted, and the results were compared with those from the backwards stepwise selection. The backwards stepwise selection was further performed separately in subgroups for each stratum of VOC period and severity of the acute infection.

In the sensitivity analysis using a 90-day interval between index date and PCC diagnosis, a full Cox regression model with all risk factors was run and compared to the main analysis (with 28-day interval), and backwards stepwise selection was performed as described. For all Cox regression models, the proportional hazards (PH) assumption was checked using graphical diagnostics and it was not violated. All analyses were conducted with STATA 18 statistical software.

### Reporting summary

Further information on research design is available in the Nature Portfolio Reporting Summary linked to this article.

## Results

From the source population, 810 851 individuals had registered COVID-19 during the study inclusion period. Among these, 11 464 (1.4%) received a PCC diagnosis during follow-up (Supplementary data 1). The time gap between the first infection and later PCC diagnosis ranged from one to 38 months with a median of 2.7 months. The majority (75%) of PCC cases got their diagnosis within 7 months after their first registered infection.

The older age groups showed higher crude incidence of PCC diagnosis (8.8 and 9.4 cases per 1000 person-years for 40–79-year-olds and ≥80-year-olds, respectively, compared to 3.2 for <40-year-olds, Supplementary data 1). The group of women compared to men, non-Swedish born individuals compared to Swedish born, and essential workers compared to other employment categories also showed higher crude incidence rates. The group of individuals with comorbidities showed higher incidence rate of PCC diagnosis compared to those with no comorbidities, except for those with dementia, where the incidence rate was lower. Getting COVID-19 during the preAlpha- and Alpha-dominated periods was associated with a higher incidence rate of PCC diagnosis compared to other VOC periods, and experiencing more severe acute COVID-19 resulted in markedly higher incidence rates. High incidence rates for the groups of individuals with a family member or a household member with PCC were also observed. Overall, the incidence rates of PCC diagnosis were similar between the two regions (6.5 and 6.1 cases per 1000 person-years for Region Stockholm and Region Västra Götaland, respectively).

**Fig. 2:**
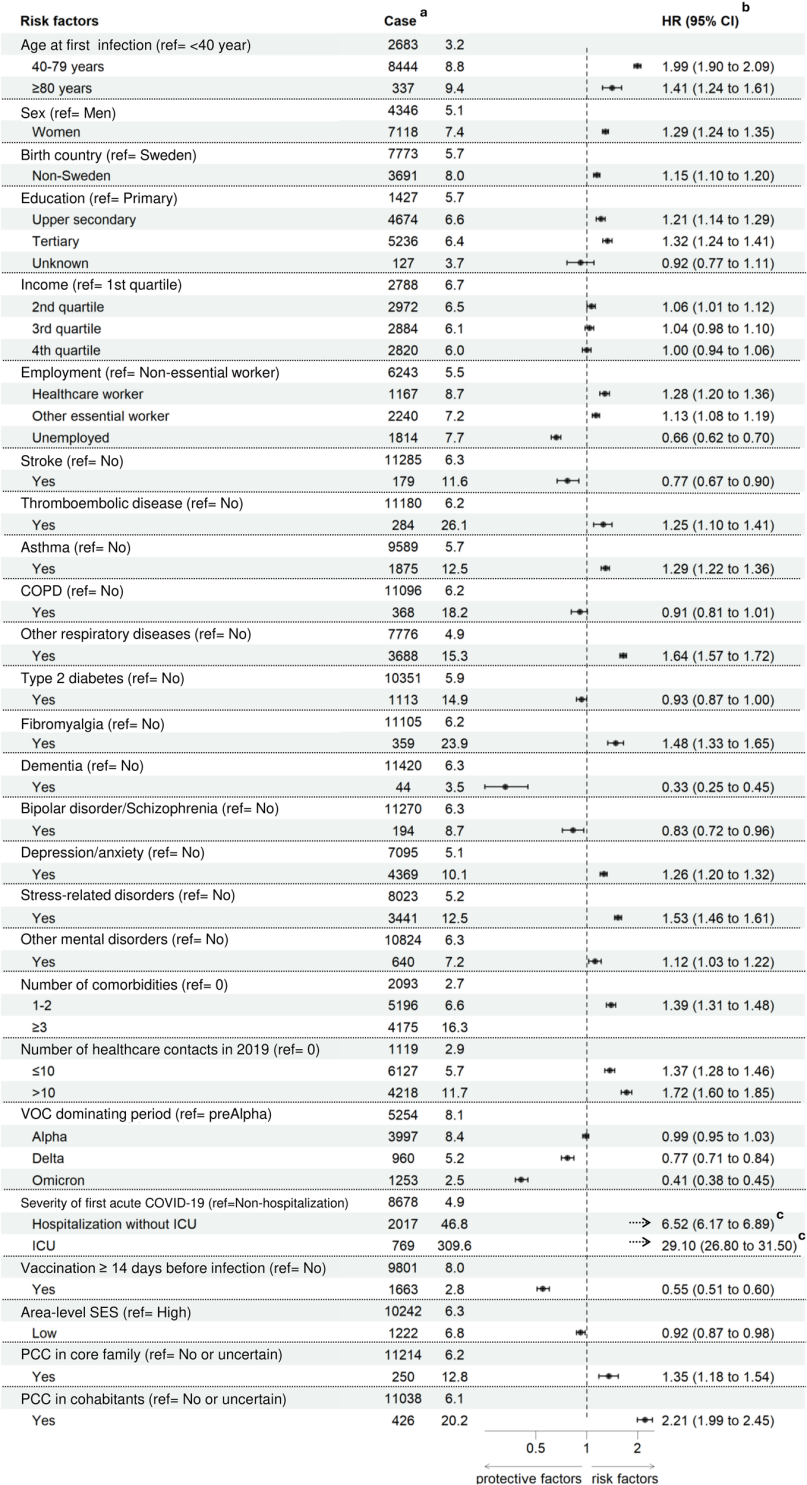
Forest plot illustrating the association between each risk factor included in the full model after backwards stepwise selection, and a valid PCC diagnosis. Study population included all individuals with a first registered COVID-19 during 1 August 2020 and 9 February 2022 (n = 810 851). Number of cases, incidence rate (IR, per 1000 person-years), adjusted hazard ratios (HR), and 95% confidence interval (95%CI) are presented. **a** Incidence rate presented as per 1000 person-years. **b** HR and 95%CI are mutually adjusted for all included variables. **c** HR and 95%CI are not shown in the figure as they largely exceed the range of the scale.

All potential risk factors selected for the study were individually, in crude unadjusted analyses, associated with the risk of receiving a PCC diagnosis after the first COVID-19 (Supplementary data 2, univariable models). When considering all 38 risk factors together, the association for some factors, especially certain comorbidities, with PCC diagnosis, became weaker (Supplementary data 2, full model, Supplementary Fig. 4). The subsequent more parsimonious multivariable regression model retained 26 variables (Supplementary data 2, multivariable model, Fig. 2). These variables were chosen based on the backwards stepwise selection, considering the robustness of the selection across different methods (Supplementary Table S3). Most of the cardiovascular comorbidities were removed, while all mental health disorders were retained. The HR estimates for retained variables from the multivariable model after backwards stepwise selection were very similar to the estimates from the full model. Among all risk factors, severity of first infection was the strongest risk factor for PCC diagnosis (HR 6.52, 95%CI 6.17-6.89; and HR 29.05, 95%CI 26.80-31.48 for hospitalization without or with ICU admission, respectively), while vaccination (any number of doses) before infection was a strong protective factor (HR 0.55, 95%CI 0.51-0.60 compared to being unvaccinated). Thromboembolism, asthma, fibromyalgia, depression/anxiety, and stress-related disorders were comorbidities associated with an increased risk of PCC, while stroke, dementia, and bipolar disorder/schizophrenia were associated with a decreased risk.

### Sub analyses and sensitivity analyses

**Fig. 3:**
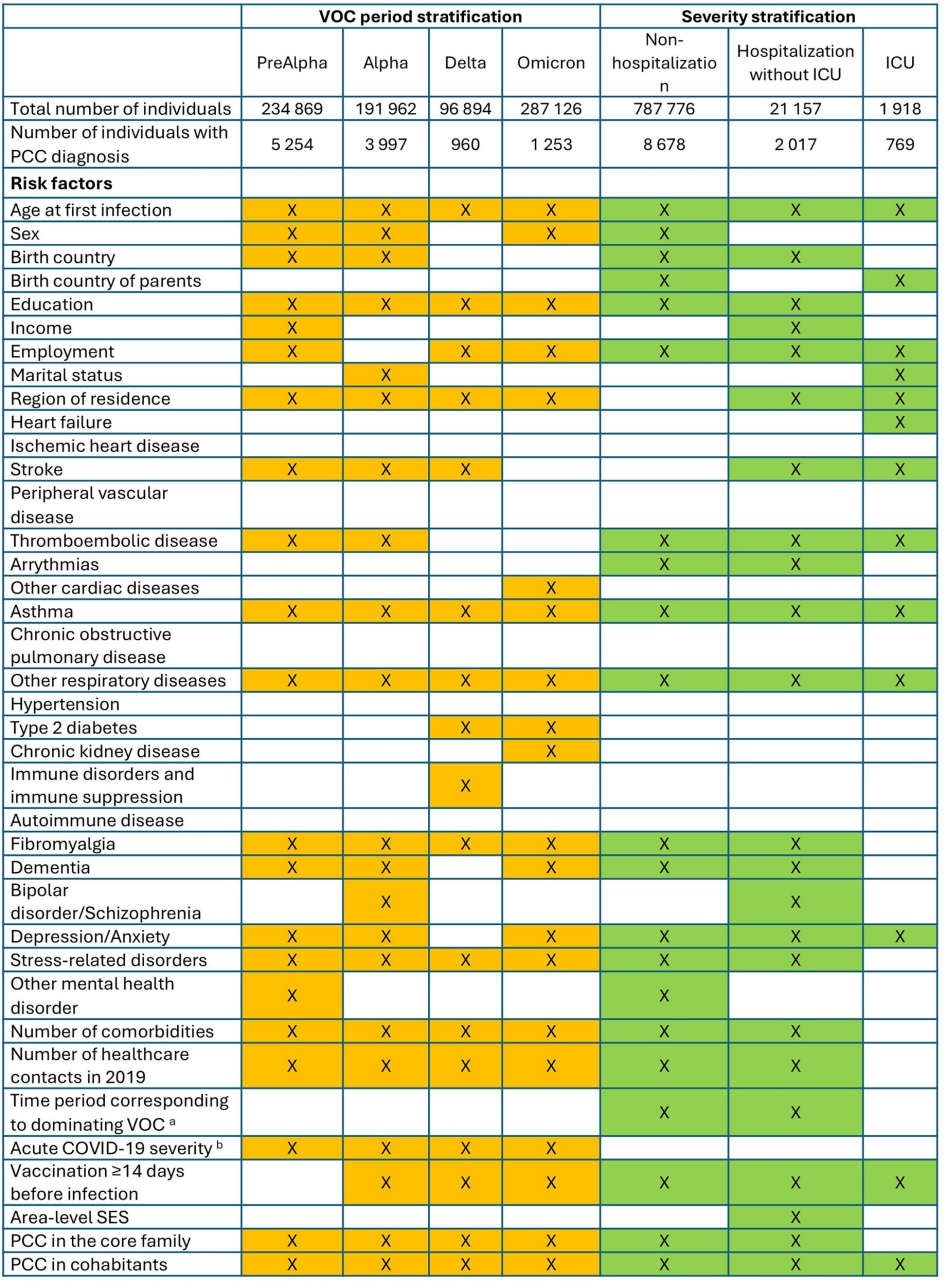
Retained variables (orange or green colored boxes with marked X) following backwards stepwise selection in separate subgroup analyses stratified by time periods dominating by variants of concern (VOC) and by severity of acute COVID-19. Analyses performed in all individuals with a first registered COVID-19 during 1 August 2020 and 9 February 2022 and with end of follow-up 30 November 2023 (n = 810 851). ^a^ VOC was not included in the VOC stratified analysis. ^b^ Acute COVID-19 severity was not included in the severity stratified analysis. PCC post-COVID-19 condition, ICU intensive care unit, SES socioeconomic status.

In the separate analyses stratified by VOC period and COVID-19 severity, there were 12 risk factors constantly selected by the model building process with PCC diagnosis across all VOC periods (Fig. 3). These were age, education level, region of residence, asthma, other respiratory diseases, fibromyalgia, stress-related disorders, number of comorbidities and healthcare contacts, acute COVID-19 severity, PCC in the core family and in cohabitants. Preexisting depression and anxiety were also considerable risk factors for most of the VOC periods, while vaccination before infection was a protecting factor during all VOC periods except the preAlpha period when it was largely unavailable (Supplementary data 3 and 4). Similarly, there were 8 risk factors constantly showing an association with PCC diagnosis across all strata of acute COVID-19 severity (Fig. 3, Supplementary data 5 and 6).

In the sensitivity analysis with 90-day (instead of 28-day) interval between index date and PCC diagnosis, the number of individuals with a valid PCC diagnosis was 8261 (1.0%). The retained risk factor set after backwards stepwise selection was quite similar to that of the main analysis using a 28-day interval, although country of birth and PCC in the core family were removed in the sensitivity analysis (Supplementary data 7).

## Discussion

In this large population-based cohort study covering 810 851 individuals, we evaluated a large set of potential risk factors for receiving a diagnosis of PCC after acute COVID-19. We show that severity of the acute disease, virus variants before Omicron, not being vaccinated, female sex, older age, being born outside of Sweden, having a higher education attainment, being a healthcare worker or another essential worker, having a family or household member with PCC, and having preexisting comorbidities such as thromboembolic disease, asthma, fibromyalgia, depression, anxiety, and stress-related disorders, were associated with increased risk of getting a diagnosis of PCC in this multivariable explorative analysis. Although most earlier studies have often been small and included selected populations, the results from a recent systematic review are mainly in accordance with the results from our study^19^. However, a novel and important empirical finding in our study is that PCC in family and cohabitants as well as being an essential worker or healthcare worker are important risk factors for receiving a diagnosis of PCC.

The strongest risk factor for PCC in this explorative study was a severe acute phase of the infection, characterized by either hospitalization or ICU admission. We have previously shown that the cumulative incidence of PCC was increased in correlation with increasing severity of the acute disease^22^. Using data from the US Department of Veterans Affairs managed care system it has been shown that the use of mechanical ventilation during the acute phase of the disease was associated with an increased risk of PCC^12^. However, distinguishing between PCC and post-intensive care syndrome can be challenging. Patients treated in ICU, regardless of reason for treatment, have a high propensity to develop long-term complications relating to the intensive treatment and ICU care rather than the disease itself^24^. It cannot be excluded that some of the diagnosed PCC cases in patients treated for their acute COVID-19 in ICU have been misclassified as PCC rather than post-intensive care syndrome. A recent study using questionnaires observed that individuals experiencing self-reported moderate or severe acute COVID-19 symptoms were at higher odds of reporting long-term symptoms^25^. In our study, we categorized individuals as either hospitalized (non-ICU or ICU) or not hospitalized. It is possible that the non-hospitalized group could be further categorized according to severity, maybe showing a higher resolution of this risk factor, however, this is not possible using Swedish register-based data.

The propensity to cause PCC for the different SARS-CoV-2 variants has previously been shown to differ, with Omicron being associated with lower risk for long-term effects^26,27^. Vaccination became increasingly common just before the Omicron period and since vaccination has been shown to decrease the risk of PCC^14^, it is important to adjust for vaccination status. In the present study, we show that being infected during the periods when Delta and Omicron were dominating was associated with a lower risk of receiving a diagnosis of PCC compared to the preAlpha variant period in a full model including vaccination status. A combination of higher immunity from vaccinations and previous infections, along with a possible lesser engagement of the lower respiratory tract by Omicron, may explain the lower risk of receiving a diagnosis of PCC from the later variants compared to preAlpha variants.

Regarding comorbidities, we show that preexisting thromboembolic disease, asthma, fibromyalgia, and common mental disorders (depression, anxiety, stress-related disorders) were associated with an increased risk of receiving a diagnosis of PCC in the multivariable model, and that preexisting stroke, dementia, and serious mental disorders (bipolar disorder, schizophrenia) were associated with a decreased risk. A study evaluating psychiatric comorbidities as risk factors for PCC in US veterans recently showed that depression, anxiety, and stress-related disorders were associated with an increased risk for PCC, but substance use and psychotic disorders were associated with a decreased risk^28^. Another study also on US veterans showed that posttraumatic stress disorder, depression, bipolar disorder, and schizophrenia were not associated with PCC^12^. Since the US veterans population is predominately male and psychiatric disorders are common, these studies do not fully reflect the general population. The large systematic review previously described noted an increased risk for PCC among individuals with preexisting depression and anxiety across the included studies^19^. In our study, individuals with serious mental disorders had a lower risk of PCC which could be due to an inherently lower risk of the condition for example related to genetic or environmental factors underlying these comorbidities that are also associated with a decreased risk for PCC. PCC could also be underdiagnosed in these patients due to for example overlapping symptoms, lower recognition of symptoms, or lower access to healthcare. In contrast, individuals with common mental disorders had a higher risk of PCC, perhaps because they are more vulnerable to certain PCC symptoms, or because these patients encounter healthcare more often and are more likely to have PCC detected. Another study showed that depression, anxiety, and stress were all associated with an increased risk of self-assessed daily life impairment after COVID-19^29^ indicating that our observed association might not only be due to increased detection of PCC by healthcare.

Female sex has previously been associated with an increased risk of PCC, which is corroborated in the present study. Since women have higher propensity to seek care for their health problems^30^, they might also be diagnosed more often. However, our analysis was adjusted for health-seeking behavior, and earlier studies using self-assessment of long-term problems have also shown a higher risk of PCC among women, indicating other mechanisms at place. For age, we show a non-linear association between age and the risk of PCC, with the highest risk in middle age, similar to what other studies have found^25^. Higher education attainment as well as being a healthcare worker were both associated with an increased risk of PCC in the analysis, while unemployment was associated with a reduced risk. This is in contrast to two other studies using self-assessed long-term symptoms which showed that longer education was associated with a decreased risk^6,31^. It is quite rare that longer education is a risk factor for disease and unemployment protective. One explanation might be that it is easier for individuals with longer education to get a diagnosis of PCC than individuals with shorter education. For example, they may be more likely to be aware of PCC and its symptoms, have easier access to care, or have lower barrier to seek healthcare, possibilities that could also explain the higher risk for healthcare workers. Individuals with shorter education or without employment might seek care more seldom, or their symptoms might be overlooked, or attributed to other conditions.

We also observed that PCC in the core family or in cohabitants were risk factors for getting a diagnosis of PCC. To the best of our knowledge, these variables have not been investigated before, although we have previously shown that the cumulative incidence of PCC for children with parents with PCC was higher than for children without^32^. PCC in the core family or in cohabitants could be seen as proxies for genetic and shared environmental factors meaning that these factors might influence the risk for PCC. This could indicate that the etiology of PCC involves genetic susceptibility, or that family and cohabitants’ experience of PCC increases awareness of long-term symptoms, which could facilitate navigation of the healthcare system.

Strengths of the present study are the inclusion of a large, population-based, and comprehensive, population of SARS-CoV-2 infected individuals, and the inclusion of individuals experiencing both mild and severe acute disease. Limitations of the study include that the diagnosis code for PCC is not yet well validated both in general and in a Swedish context. However, a Swedish study evaluated healthcare use after COVID-19 in individuals with and without a diagnosis of PCC and showed that the PCC group had significantly more healthcare contacts after the infection^11^. We show that 1.4% of all individuals with registered COVID-19 in the present study later receive a diagnosis of PCC, this is a bit lower than the most recent estimate of the prevalence of PCC from WHO^33^. Therefore, we believe that the specificity of the diagnosis code might be good, while its sensitivity remains less clear. One should be aware that the study evaluates both the risk factors for PCC itself and the risk factors for accessing healthcare and getting a diagnosis of PCC since the diagnosis is set after a clinical assessment from a physician. It should also be noted that this study explored the risk factors for getting a PCC diagnosis in a population with registered COVID-19. It was based on the two assumptions that a previous infection is a prerequisite to set a valid PCC diagnosis and that all infected individuals do not develop PCC afterwards. This approach is a way to minimize misclassifications of PCC in the register data. However, this approach means that we cannot preclude that the observed risk factors for PCC may also be associated with similar symptom patterns in individuals who were not infected with SARS-CoV-2. Since only infected individuals were included in the study, the identified risk factors should be interpreted as associated with receiving a PCC diagnosis after COVID-19, rather than as specific predictors of symptom development uniquely attributable to SARS-CoV-2 infection. This distinction is important, as similar symptoms could potentially occur in uninfected individuals due to other causes. Furthermore, despite our large set of evaluated risk factors, we unfortunately did not have access to clinical features of the acute infection (biomarkers, antibodies, viral load etc.). Lastly, in a multiple risk factor analysis, the risk for false-positive findings could be increased due to multiple testing, as well as a risk for “Table 2 fallacy”^34^ since the correct and complete set of confounding variables for each of the variables might not be the same and might not be included in the model. However, as this is an exploratory study of multiple etiologic factors it is important to acknowledge that there needs to be independent confirmation of the findings, with focus on one risk factor at a time.

In conclusion, we have conducted an explorative risk factor analysis simultaneously evaluating multiple risk factors for receiving a diagnosis of PCC in a large and comprehensive Swedish population. In this analysis, we show that the most important risk factors for getting a diagnosis of PCC after confirmed infection in a multivariable model setting are severe acute infection, infection earlier in the study period, not being vaccinated, female sex, older age, higher education, being an essential worker, comorbidities such as common mental disorders and asthma, as well as having a biological family member or a household member also diagnosed with PCC. The present findings could serve as a basis for future targeting of preventive measures of PCC but also highlight the need for future research especially for disentangling the familial and household risks of PCC.

## Supplementary information

Supplementary material

Description of additional supplementary files

Supplementary data 1

Supplementary data 2

Supplementary data 3

Supplementary data 4

Supplementary data 5

Supplementary data 6

Supplementary data 7

Reporting Summary

## Data Availability

The data used in this study are pseudonymized individual-level data from Swedish healthcare registers and can be obtained after application to the respective Swedish public data holders (National Board of Health and Welfare, Statistics Sweden, Swedish Public Health Agency) on the basis of ethics approval for the research in question (from the Swedish Ethical Review Authority https://etikprovningsmyndigheten.se/), subject to relevant legislation, processes, and data protection (for more information email: registrator@etikprovning.se).
