# Supplementary material for: Exploring predictors of post-COVID-19 condition among 810 851 individuals in Sweden
Source: Commun Med (Lond). 2025 Oct 30;5:445. doi: 10.1038/s43856-025-01157-2 (PMC12575654; doi:10.1038/s43856-025-01157-2)

Supplementary material

Exploring predictors of post-COVID-19 condition among 810 851 individuals in Sweden

Yiyi Xu, Huiqi Li, Robert Sigström, Lisa Lundberg-Morris, Magnus Gisslén, Simon Larsson, Fredrik Nyberg, Maria Bygdell

Contents

Supplementary tables 3

Supplementary table S1. Definitions of the comorbidities used in the risk factors analysis for post-COVID-19 condition. 3

Supplementary table S2. Definitions of the periods when different virus variants where dominating. 4

Supplementary table S3. Retained variables following LASSO selection and backwards stepwise selection. 5

Supplementary figures 6

Supplementary figure S1. The non-linear association between age and post-COVID-19 condition (PCC). 6

Supplementary figure S2. The non-linear association between number of healthcare contacts in 2019 and post-COVID-19 condition (PCC). 7

Supplementary figure S3. The non-linear association between area-level socioeconomic status (SES) and post-COVID-19 condition (PCC). 8

Supplementary figure S4. Forest plot illustrating the association between each risk factor from the full model and a valid PCC diagnosis. 9

Supplementary tables

Supplementary table S1. Definitions of the comorbidities used in the risk factors analysis for post-COVID-19 condition.

Comorbidity	ICD-10-SE diagnosis code	ATC code
Heart failure	I50	
Ischemic heart disease	I20-25, Z951, Z955	
Stroke	I61-64	
Peripheral vascular disease	I70-71, I73.1, I73.8-9, I77.1, I79.0, I79.2, K55.1, K55.8-9, Z958-9	
Thromboembolic disease	I82.2-3, I82.8-9, I26	
Arrythmias	I441, I442, I452, I453, I456, I459, I46-48, I490, I495	
Other cardiac diseases	I05-09, I26-28, I32-43, I51, R001, R011, Q20-28	
Asthma	J45	
Chronic obstructive pulmonary disease	J44	
Other respiratory diseases	J09-22, J40-99 (without J44 and J45)	
Hypertension	I10-15	C07 (except C07AA07), C02, C03A, C03EA01, C08C, C09A-X
Type 2 diabetes	E11	
Chronic kidney disease	N18	
Immune disorders and immune suppression	D80-84	L04A
Autoimmune disease	D51.0, D59.0, D59.1, D69.3, E03.9, E06.3, E05.0, E05.9, E06, E10, G35, G61.0, G70.0, H20, H46.9, I00–02, I06, I09, I01.0–2, I01.8–9, I02.0, I02.9, I05.1, I06.0–2, I02.9, I05.1, I06.0–2, I02.9, I05.1, I06.0–2, I06.8–9, I09.0–2, I09.8–9, K50, K51, K52.3, K52.8, K52.9, K52.9W, K73, K74.3, K90.0, L10, L12, L40, L63, L80.9, M02.3, M05, M06, M08.0, M08.2, M08, M30-M36, M31.3, M31.5-6, M35.3, M32.1, M32.9, M33, M34, M35.0, M45.9	
Fibromyalgia	M79.7	
Dementia	F00-03	
Bipolar disorder/Schizophrenia	F20-29, F31	
Depression/Anxiety	F32, F33, F40-42	
Stress-related disorders	F43	
Other mental health disorders	F00-99 (without F00-03, F20-29, F31-33, F40-43)	

ICD-10-SE=international classification of diseases 10th revision Swedish version, ATC=anatomical therapeutic chemical classification system.

Supplementary table S2. Definitions of the periods when different virus variants where dominating.
From the start of the pandemic and to the end of follow-up of the present study.

**Virus variant**	**Period start**	**Period end**
PreAlpha	1 February 2020	31 January 2021
Alpha	1 February 2021	30 June 2021
Delta	1 July 2021	31 December 2021
Omicron	1 January 2022	30 November 2023

Supplementary table S3. Retained variables following LASSO selection and backwards stepwise selection. Differences highlighted by bold text.

**LASSO— BIC chosen variable**	**LASSO— Adaptive chosen variable**	**Backwards selection**
Age at first infection	Age at first infection	Age at first infection
Sex	Sex	Sex
Country of birth	Country of birth	Country of birth
	**Parents' country of birth**	
Education	Education	Education
Income	Income	Income
Employment	Employment	Employment
Stroke	Stroke	Stroke
Thromboembolic disease	Thromboembolic disease	Thromboembolic disease
**Arrythmias**	**Arrythmias**	
Asthma	Asthma	Asthma
COPD	COPD	COPD
Other respiratory diseases	Other respiratory diseases	Other respiratory diseases
Hypertension	**Hypertension**	Hypertension
Type 2 diabetes	Type 2 diabetes	Type 2 diabetes
**Chronic kidney disease**		
	**Autoimmune diseases**	
Fibromyalgia	Fibromyalgia	Fibromyalgia
Dementia	Dementia	Dementia
Bipolar disorder/Schizophrenia	Bipolar disorder/Schizophrenia	Bipolar disorder/Schizophrenia
Depression/anxiety	Depression/anxiety	Depression/anxiety
Stress-related disorders	Stress-related disorders	Stress-related disorders
Other mental disorders	Other mental disorders	Other mental disorders
Number of comorbidities	Number of comorbidities	Number of comorbidities
Number of healthcare contacts in 2019	Number of healthcare contacts in 2019	Number of healthcare contacts in 2019
VOC dominating period	VOC dominating period	VOC dominating period
Severity of first acute COVID-19	Severity of first acute COVID-19	Severity of first acute COVID-19
Vaccination 14 days before infection	Vaccination 14 days before infection	Vaccination 14 days before infection
Area-level SES	Area-level SES	Area-level SES
PCC in core family	PCC in core family	PCC in core family
PCC in cohabitants	PCC in cohabitants	PCC in cohabitants

Supplementary figures

Supplementary figure S1. The non-linear association between age and post-COVID-19 condition (PCC).
Including all individuals with a first registered COVID-19 between 1 August 2020 and 9 February 2022 (n=810 851).

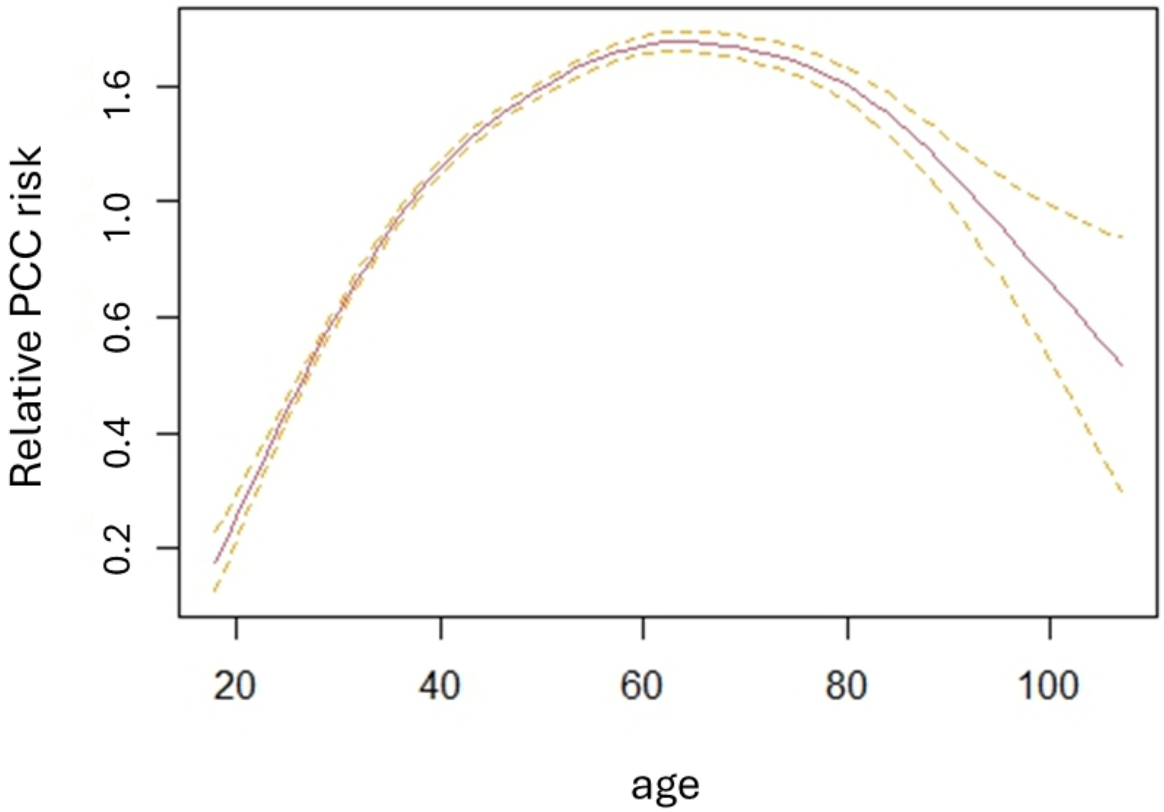

Supplementary figure S2. The non-linear association between number of healthcare contacts in 2019 and post-COVID-19 condition (PCC). Including all individuals with a first registered COVID-19 between 1 August 2020 and 9 February 2022 (n=810 851).

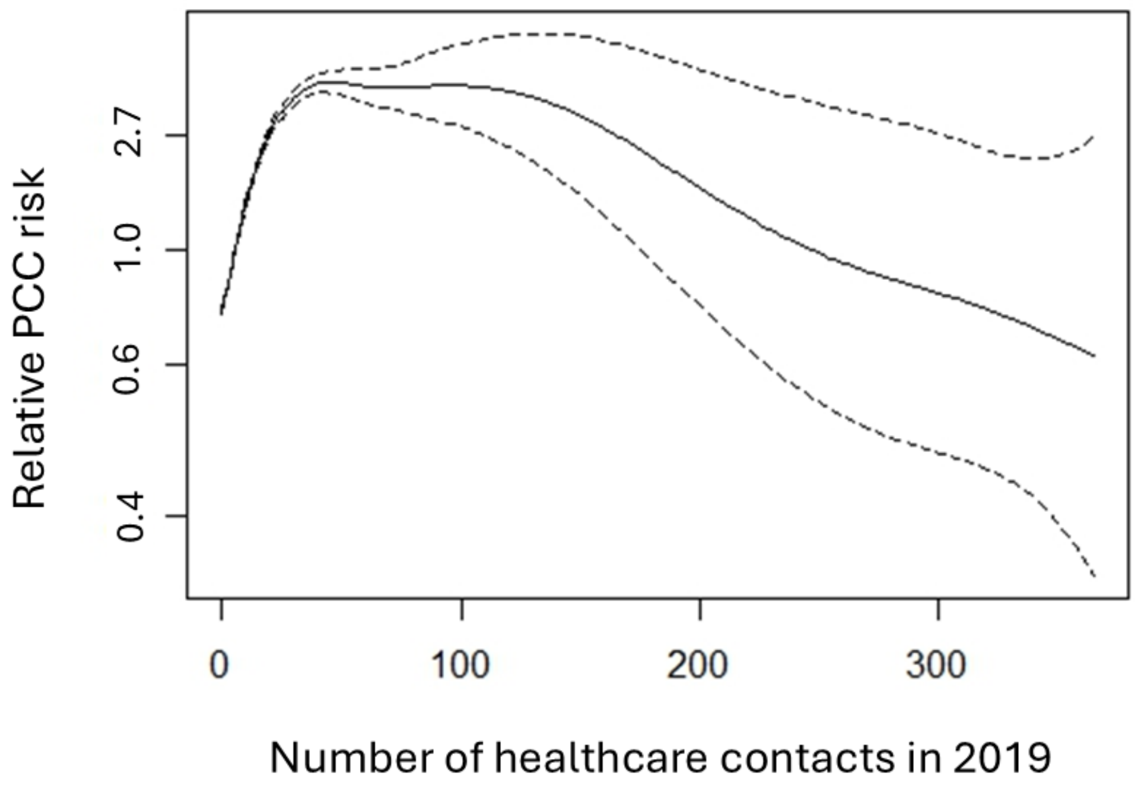

Supplementary figure S3. The non-linear association between area-level socioeconomic status (SES) and post-COVID-19 condition (PCC). Including all individuals with a first registered COVID-19 between 1 August 2020 and 9 February 2022 (n=810 851).

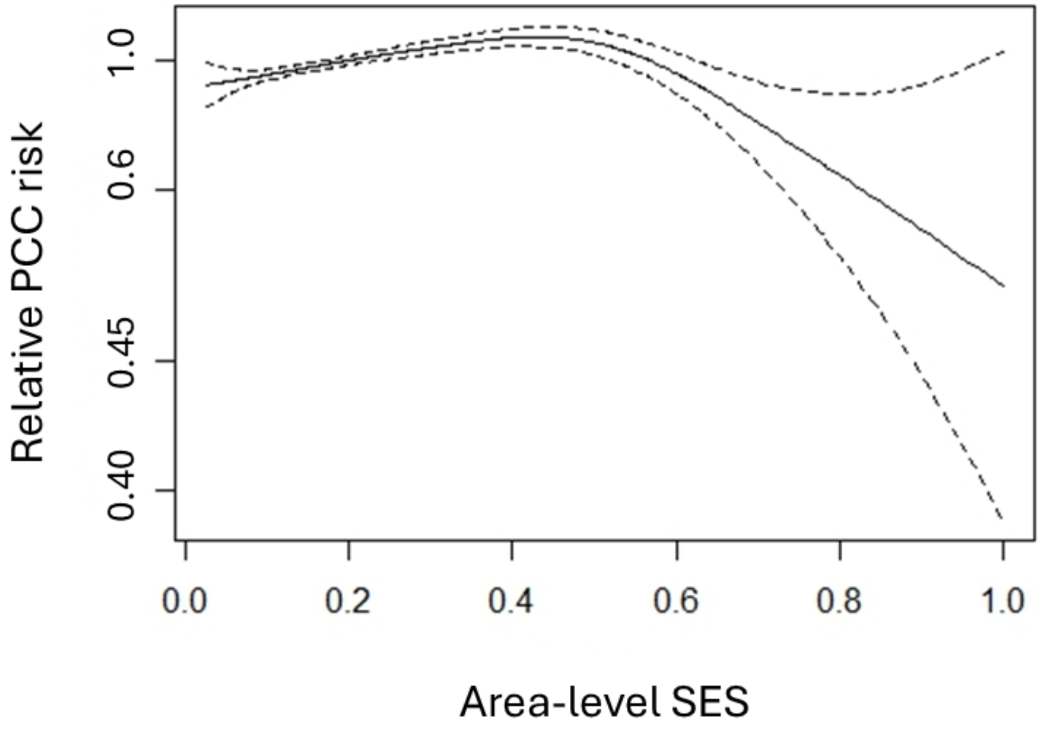

Supplementary figure S4. Forest plot illustrating the association between each risk factor from the full model and a valid PCC diagnosis. Including all individuals with a first registered COVID-19 during 1 August 2020 and 9 February 2022 (n=810 851). Number of cases, incidence rate (IR, per 1000 person-years), hazard ratios (HR), and 95% confidence interval (95%CI) are presented. Source data are presented in Supplementary data 2.

[a] Incidence rate presented as per 1000 person-years.

[b] HR and 95%CI are mutually adjusted for all included variables.

[c] HR and 95%CI are not shown in the figure as they largely exceed the range of the scale.

Risk factors	Case [a]		HR (95% CI) [b]
Age at first infection (ref= <40 year)	2683	3.2	
40-79 years	8444	8.8	1.99 (1.89 to 2.09)
≥80 years	337	9.4	1.43 (1.25 to 1.64)
Sex (ref=Men)	4346	5.1	
Women	7118	7.4	1.29 (1.24 to 1.35)
Birth country (ref=Sweden)	7773	5.7	
Non-Sweden	3691	8.0	1.15 (1.05 to 1.26)
Birth country of parents (ref=Both from Sweden)	6092	5.7	
One from Sweden	1073	6.3	1.04 (0.97 to 1.11)
Both from non-Sweden	1329	5.6	0.96 (0.89 to 1.04)
Unknown	2970	8.6	1.01 (0.92 to 1.12)
Education (ref=Primary)	1427	5.7	
Upper secondary	4674	6.6	1.21 (1.14 to 1.29)
Tertiary	5236a	6.4	1.32 (1.24 to 1.41)
Unknown	127	3.7	0.92 (0.76 to 1.10)
Income (ref=1st quartile)	2788	6.7	
2nd quartile	2972	6.5	1.06 (1.01 to 1.12)
3rd quartile	2884	6.1	1.04 (0.98 to 1.10)
4th quartile	2820	6.0	1.00 (0.94 to 1.07)
Employment (ref=Non-essential worker)	6243	5.5	
Healthcare worker	1167	8.7	1.28 (1.20 to 1.36)
Other essential worker	2240	7.2	1.13 (1.08 to 1.19)
Unemployed	1814	7.7	0.66 (0.62 to 0.71)
Marital status (ref=Married)	5439	7.3	
Not married	6025	5.6	0.99 (0.95 to 1.03)
Region of residence (ref=Stockholm)	6328	6.5	
Västra Götaland	5136	6.1	1.02 (0.98 to 1.06)
Heart failure (ref=No)	11118	6.2	
Yes	346	18.0	0.98 (0.87 to 1.12)
Ischemic heart disease (ref=No)	10925	6.1	
Yes	539	14.4	0.99 (0.90 to 1.09)
Stroke (ref=No)	11285	6.3	
Yes	179	11.6	0.78 (0.67 to 0.90)
Peripheral vascular disease (ref=No)	11265	6.2	
Yes	199	16.2	1.00 (0.86 to 1.16)
Thromboembolic disease (ref=No)	11180	6.2	
Yes	284	26.1	1.21 (1.05 to 1.40)
Arrhythmias (ref=No)	10854	6.1	
Yes	610	13.2	0.94 (0.86 to 1.04)
Other cardiac diseases (ref=No)	10754	6.1	
Yes	710	15.1	1.02 (0.93 to 1.13)
Asthma (ref=No)	9589	5.7	
Yes	1875	12.5	1.27 (1.20 to 1.34)
COPD (ref=No)	11096	6.2	
Yes	368	18.2	0.91 (0.82 to 1.02)
Other respiratory diseases (ref=No)	7776	4.9	
Yes	3688	15.3	1.62 (1.54 to 1.70)
Hypertension (ref=No)	8109	5.3	
Yes	3355	11.9	0.96 (0.91 to 1.01)
Type 2 diabetes (ref=No)	10351	5.9	
Yes	1113	14.9	0.94 (0.87 to 1.01)
Chronic kidney disease (ref=No)	11182	6.2	
Yes	282	16.5	0.96 (0.85 to 1.09)
Immune disorders and immune suppression (ref=No)	11114	6.2	
Yes	350	12.1	1.03 (0.92 to 1.15)
Auto immune disease (ref=No)	9360	5.8	
Yes	2104	9.9	0.95 (0.90 to 1.01)
Fibromyalgia (ref=No)	11105	6.2	
Yes	359	23.9	1.47 (1.32 to 1.64)
Dementia (ref=No)	11420	6.3	
Yes	44	3.5	0.33 (0.25 to 0.45)
Bipolar disorder/Schizophrenia (ref=No)	11270	6.3	
Yes	194	8.7	0.83 (0.71 to 0.95)
Depression/anxiety (ref=No)	7095	5.1	
Yes	4369	10.1	1.24 (1.18 to 1.30)
Stress-related disorders (ref=No)	8023	5.2	
Yes	3441	12.5	1.51 (1.43 to 1.59)
Other mental disorders (ref=No)	10824	6.3	
Yes	640	7.2	1.10 (1.01 to 1.20)
Number of comorbidities (ref=0)	2093	2.7	
1-2	5196	6.6	1.43 (1.34 to 1.53)
≥3	4175	16.3	1.62 (1.45 to 1.80)
Number of healthcare contacts in 2019 (ref=0)	1119	2.9	
≤10	6127	5.7	1.37 (1.28 to 1.47)
>10	4218	11.7	1.72 (1.60 to 1.86)
VOC dominating period (ref=preAlpha)	5254	8.1	
Alpha	3997	8.4	0.99 (0.95 to 1.03)
Delta	960	5.2	0.78 (0.71 to 0.84)
Omicron	1253	2.5	0.41 (0.38 to 0.45)
Severity of first acute COVID-19 (ref=Non-hospitalization)	8678	4.9	
Hospitalization without ICU	2017	46.8	6.57 (6.22 to 6.94) [c]
ICU	769	309.6	29.27 (27.00 to 31.74) [c]
Vaccination ≥14 days before infection (ref=No)	9801	8.0	
Yes	1663	2.8	0.55 (0.51 to 0.60)
Area-level SES (ref=High)	10242	6.3	
Low	1222	6.8	0.92 (0.87 to 0.98)
PCC in core family (ref=No or uncertain)	11214	6.2	
Yes	250	12.8	1.35 (1.19 to 1.55)
PCC in cohabitants (ref=No or uncertain)	11038	6.1	
Yes	426	20.2	2.20 (1.99 to 2.44)

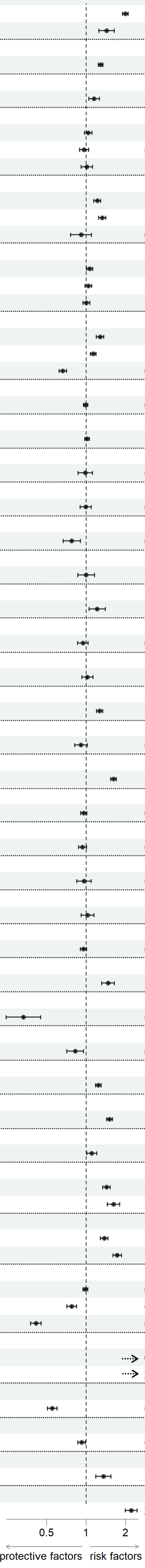

Supplement: Supplementary file 1 — Supplementary material [file 43856_2025_1157_MOESM1_ESM.pdf]
