# Supplementary material for: Exploring predictors of post-COVID-19 condition among 810 851 individuals in Sweden
Source: Commun Med (Lond). 2025 Oct 30;5:445. doi: 10.1038/s43856-025-01157-2 (PMC12575654; doi:10.1038/s43856-025-01157-2)
Supplement: Supplementary file 2 — Description of additional supplementary files [file 43856_2025_1157_MOESM2_ESM.pdf]

Description of Additional Supplementary Files

File name: Supplementary Data 1
Description: Descriptive analysis of included baseline selected potential risk factors for post-COVID-19 condition (PCC). Including all individuals with a first registered COVID-19 during 1 August 2020 and 9 February 2022. Data are presented as counts and percent in the total cohort and by PCC diagnosis at end of follow-up (30 November 2023), and additionally incidence rates of PCC (per 1000-person-years) in the PCC group for each category of risk factors.

File name: Supplementary Data 2
Description: HR and 95%CI for risk factors from single-variable models, a joint full model, and a multivariable model for the risk of post-COVID-19 condition. Following backward stepwise selection from the full model until end of follow-up 30 November 2023 in all individuals with a first registered COVID-19 during 1 August 2020 and 9 February 2022.

File name: Supplementary Data 3
Description: Descriptive analysis of the included risk factors for post-COVID-19 condition (PCC) diagnosis. Including all individuals with a first registered COVID-19 during 1 August 2020 and 9 February 2022, divided by VOC strata. Data are presented as counts (n), percents (%), and incidence rates (IR, per 1000-person-years) in the total cohort and in PCC cases.

File name: Supplementary Data 4
Description: Risk factor analysis of the included risk factors for post-COVID-19 condition (PCC) diagnosis. Including all individuals with a first registered COVID-19 during 1 August 2020 and 9 February 2022, divided by VOC strata. HR and 95%CI are presented for the retained risk factors in the multivariable model following backward stepwise selection.

File name: Supplementary Data 5
Description: Descriptive analysis of the included risk factors for post-COVID-19 condition (PCC) diagnosis. Including all individuals with a first registered COVID-19 during 1 August 2020 and 9 February 2022, divided by acute COVID-19 severity strata. Data are presented as counts (n), percents (%), and incidence rates (IR, per 1000-person-years) in the total cohort and in PCC cases.

File name: Supplementary Data 6
Description: Risk factor analysis of the included risk factors for post-COVID-19 condition (PCC) diagnosis. Including all individuals with a first registered COVID-19 during 1 August 2020 and 9 February 2022, divided by acute COVID-19 severity. HR and 95%CI are presented for the retained risk factors in the multivariable model following backward stepwise selection.

File name: Supplementary Data 7

Description: Risk factor analysis of the included risk factors for post-COVID-19 condition (PCC) diagnosis. Including all individuals with a first registered COVID-19 during 1 August 2020 and 9 February 2022, using a 90-day interval between registered infection and PCC diagnosis. HR and 95%CI are presented for the full model and the retained risk factors in the multivariable model following backward stepwise selection.
